# Unusual Presentation of Meckel's Diverticulitis Causing Small Bowel Obstruction Masquerading as Appendicitis

**DOI:** 10.7759/cureus.70293

**Published:** 2024-09-26

**Authors:** Manish Babbu UG, Shanthini Vaitheeswaran, Khalilur Rahman A, Vijayshree Shyam Sundar, Balavignesh Durai

**Affiliations:** 1 Department of General Surgery, Saveetha Medical College and Hospital, Saveetha Institute of Medical and Technical Sciences, Saveetha University, Chennai, IND

**Keywords:** appendicitis, case report, meckel's diverticulum, small bowel obstruction, torsion abnormality

## Abstract

Meckel's diverticulum (MD) is a prevalent congenital abnormality of the gastrointestinal tract. While it may not show any symptoms, it has the potential to cause serious complications, such as intestinal obstruction. This case report presents a case of a 27-year-old male who presented to the emergency department with migrating right lower abdomen pain and vomiting. An initial diagnosis of acute appendicitis was made. An erect X-ray of the abdomen showed features of small bowel obstruction and with a clinical suspicion of Meckel's, a diagnostic laparoscopy had been planned. However, the diagnostic laparoscopy identified a gangrenous MD with axial torsion, with an ileal loop knotting at the base of Meckel's, causing small bowel obstruction. This entanglement led to an obstruction, which is a rare and challenging clinical scenario. Surgical resection of the affected bowel segment, including the MD, was performed, leading to a complete recovery of the patient. This case study emphasizes the diagnostic difficulties presented by MD, particularly when its symptoms resemble more prevalent illnesses like appendicitis. The rare incidence of axial torsion resulting in gangrene in MD with small bowel obstruction highlights the significance of including this illness in the differential diagnosis of acute abdomen.

## Introduction

Meckel's diverticulum (MD) was first documented by Fabricius Hildanus in 1598 [1]. However, it has been designated with the name of Johann Friedrich Meckel, who first identified its embryonic origin in 1809 [2]. MD is a congenital condition that occurs when the vitelline or omphalomesenteric duct fails to completely close during embryonic development [3].

It is one of the predominant congenital anomalies of the gastrointestinal system, affecting approximately 1-3% of the population [4]. MD is commonly asymptomatic and is frequently detected incidentally during surgical procedures or diagnostic imaging for other conditions [5]. However, it can also lead to complications such as hemorrhage, inflammation, and obstruction [6].

Gastrointestinal bleeding caused by ulceration due to acid formed by heterotopic gastric mucosa is the most prevalent complication of MD in the pediatric population [7]. On the contrary, intestinal obstruction is the most common complication among adult patients. Intestinal obstruction can arise by various mechanisms, such as the presence of an omphalomesenteric band, internal hernia through vitelline duct remnants, volvulus encompassing remnants of the vitelline duct, intussusception, incarceration within a hernia sac (known as Littre's hernia), or chronic Meckel's diverticulitis. Intestinal obstruction that can occur in adults due to the presence of MD remains a clinical challenge [8].

The case report depicted here has a small bowel obstruction that was not because of the usual mechanisms. The occurrence of axial torsion around the small base of MD is an extremely rare complication and remarkably unusual in literature. This leads to a compromise in the blood flow, which in turn causes gangrene of the MD [9]. This article reports a rare scenario, wherein there is an axial rotation of the MD, along with ileal loop knotting at the base, which is the lead point for small bowel obstruction.

## Case presentation

A 27-year-old gentleman presented to the emergency room with a two-day history of migratory pain in the right lower abdomen associated with two episodes of vomiting. There were no prior instances of such symptoms in his medical history. Upon physical examination, tenderness was observed in the right iliac fossa, left iliac fossa, and hypogastric regions. Signs of peritonitis were evident, as indicated by a pulse rate of 104 beats per minute. Routine investigations were done, and the patient was found to have leukocytosis with an associated shift to the left, as shown in Table 1.

**Table 1:** Blood investigations showing elevated total leukocyte count and neutrophilia. g: grams; dL: deciliters; %: percentage; cumm: cubic millimeter; mEq: milliequivalent; L: liter.

S. No	Test	Values	Units	Reference range
1	Hemoglobin	15.6	g/dl	13-16 g/dl
2	Total leukocyte count	14,570	lakhs/cumm	4,000-10,000 lakhs/cumm
3	Neutrophils	88.8	%	40-80%
4	Lymphocytes	9.1	%	20-40%
5	Monocytes	1.5	%	2-10%
6	Eosinophils	0.3	%	1-6%
7	Basophils	0.3	%	1-2%
8	Platelet count	3.88	lakhs/cumm	1.5-4.5 lakhs/cumm
9	Serum sodium	137	mEq/L	137-145 mEq/L
10	Serum potassium	5	mEq/L	3.5-5 mEq/L
11	Serum chloride	101	mEq/L	96-106 mEq/L
12	Serum bicarbonate	24.2	mEq/L	22-26 mEq/L

**Figure 1:**
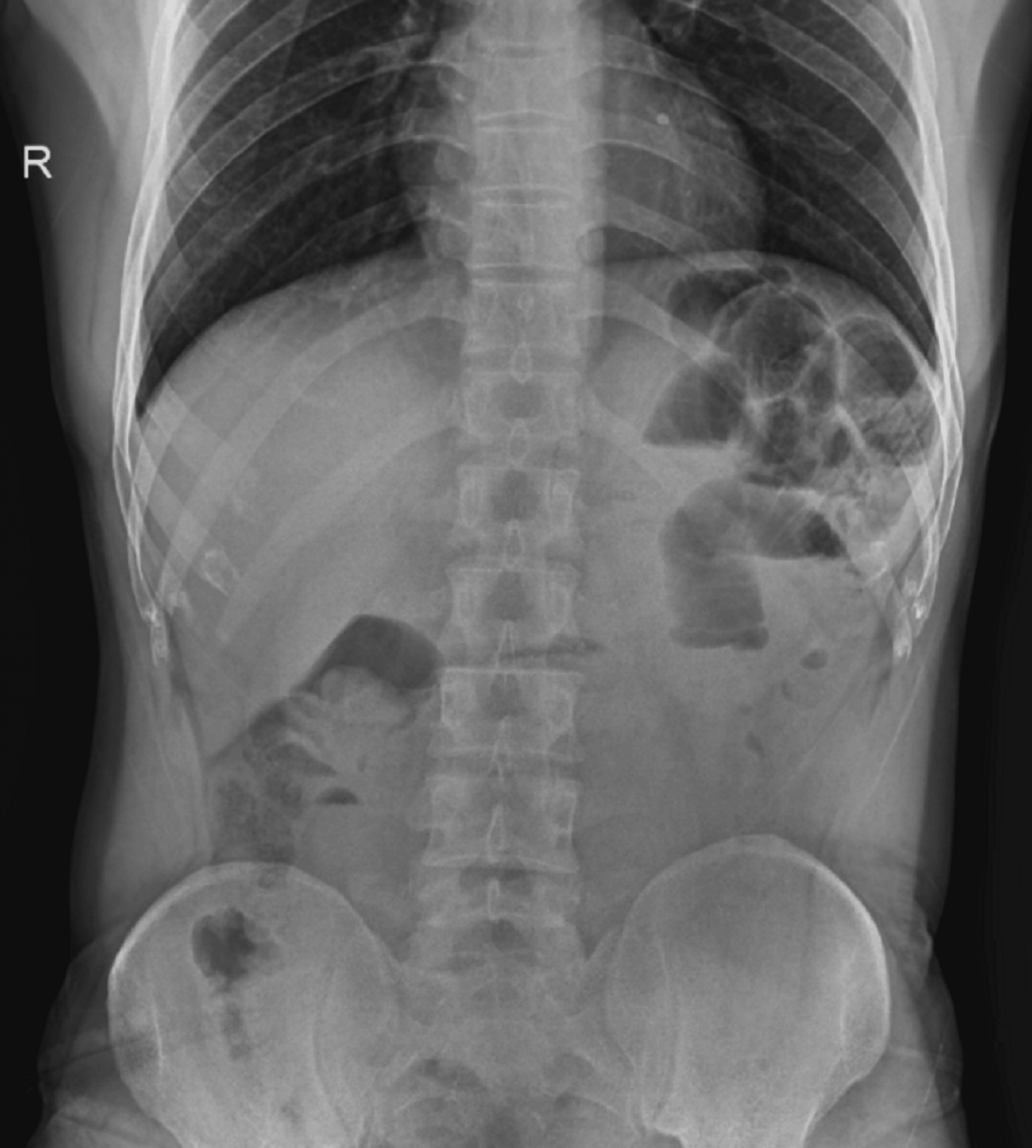
Erect X-ray of the abdomen showing multiple air fluid levels suggesting small bowel obstruction.

 Multiple air-fluid levels were noted in the erect X-ray of the abdomen, as shown in Figure 1.

**Figure 2:**
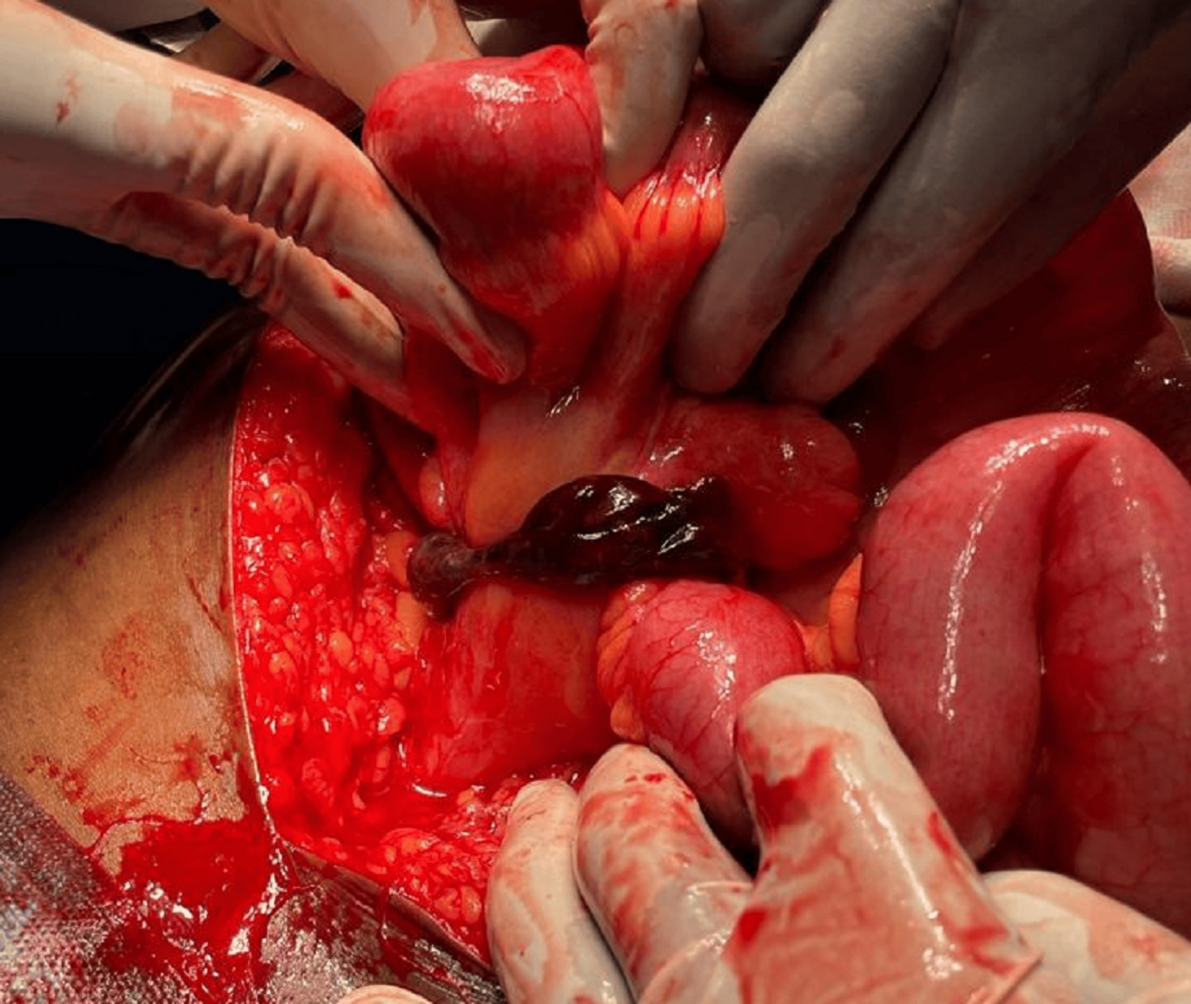
Gangrenous Meckel's diverticulum found intraoperatively prior to unwinding of the bowel loops at the base.

USG of the abdomen showed a well-defined blind-ending cystic tubular structure approximately 20 mm in diameter, which was non-compressible and peristaltic in the right iliac region, likely connected to the cecum. Additionally, mild free fluid was observed in the right iliac fossa, suggesting the possibility of acute appendicitis. The patient was initially diagnosed with acute appendicitis with small bowel obstruction and proceeded with diagnostic laparoscopy after receiving initial resuscitation. During the surgical procedure, it was discovered that the patient had a gangrenous MD that was causing small bowel obstruction, as observed in Figure 2.

**Figure 3:**
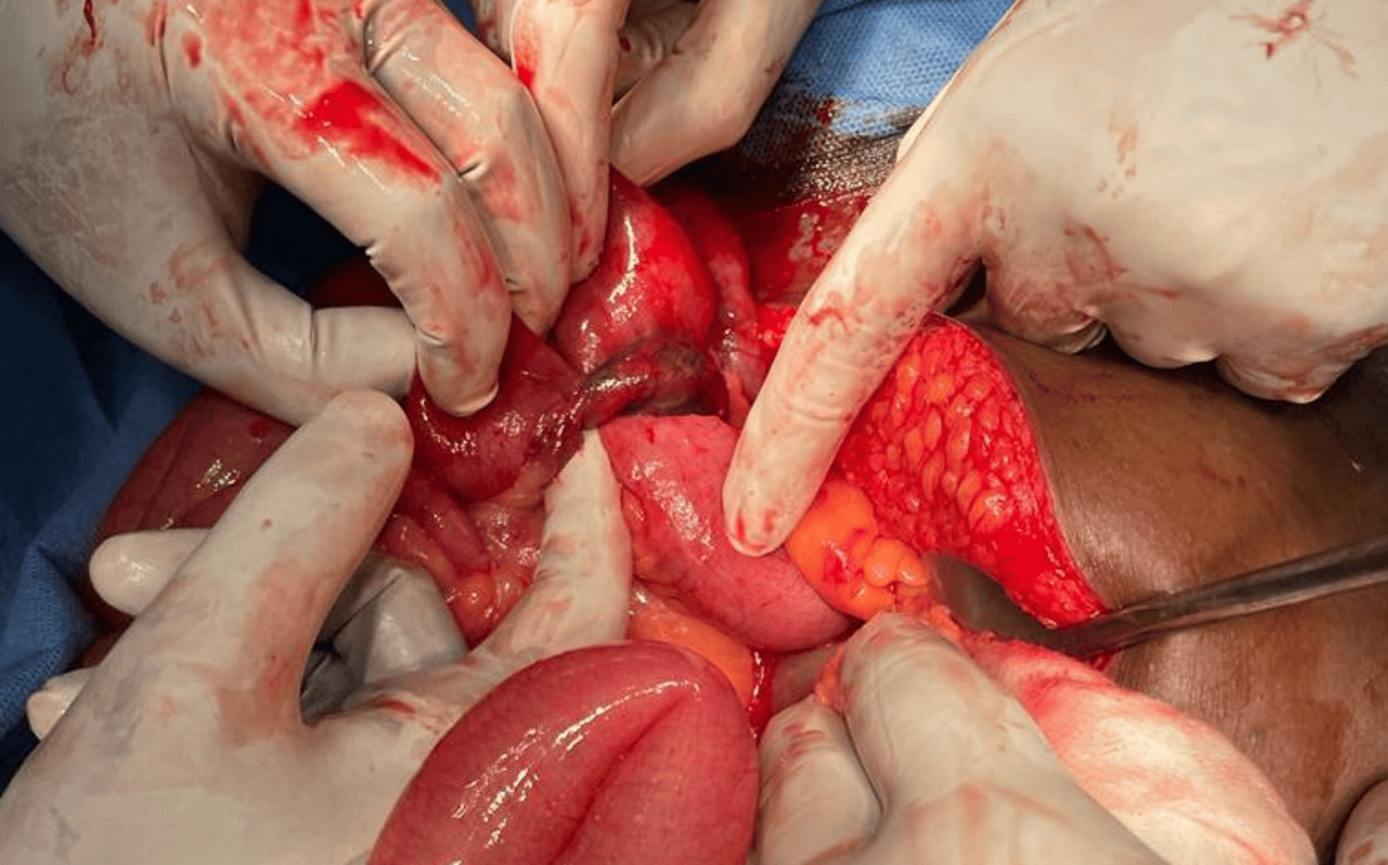
Ileal loop seen encircling the Meckel's Diverticulum, thereby compromising the vascularity of the Meckel's diverticulum.

The long MD had undergone axial rotation with ileal loop knotting at the base, as witnessed in Figure 3.

**Figure 4:**
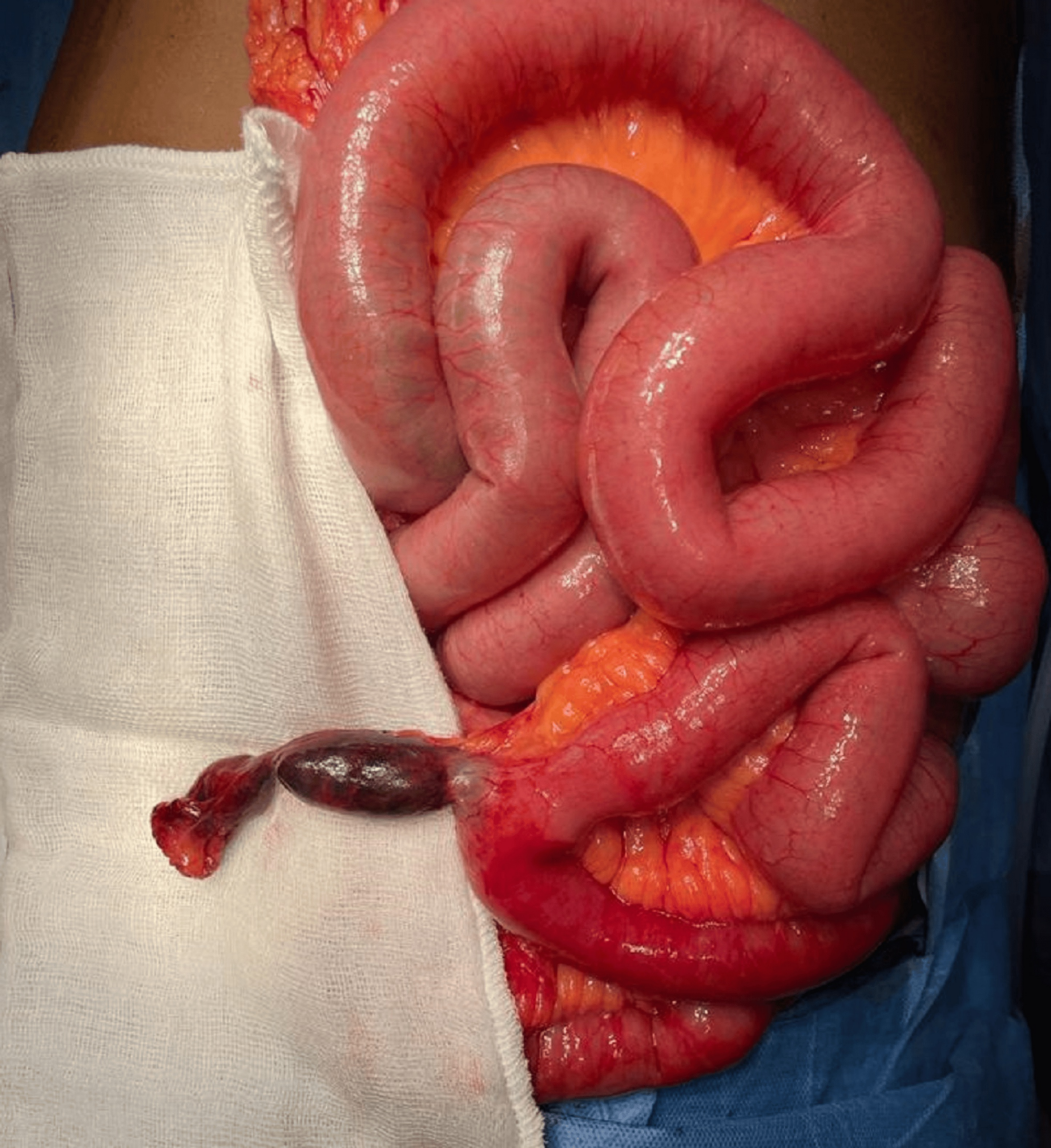
Encountered diverticulum that was untwisted and placed prior to resection.

The affected part of the bowel, along with the MD, was resected, and the small bowel was anastomosed end-to-end in a double-layered fashion with a wash given. De-rotated gangrenous MD prior to resection is shown in Figure 4.

**Figure 5:**
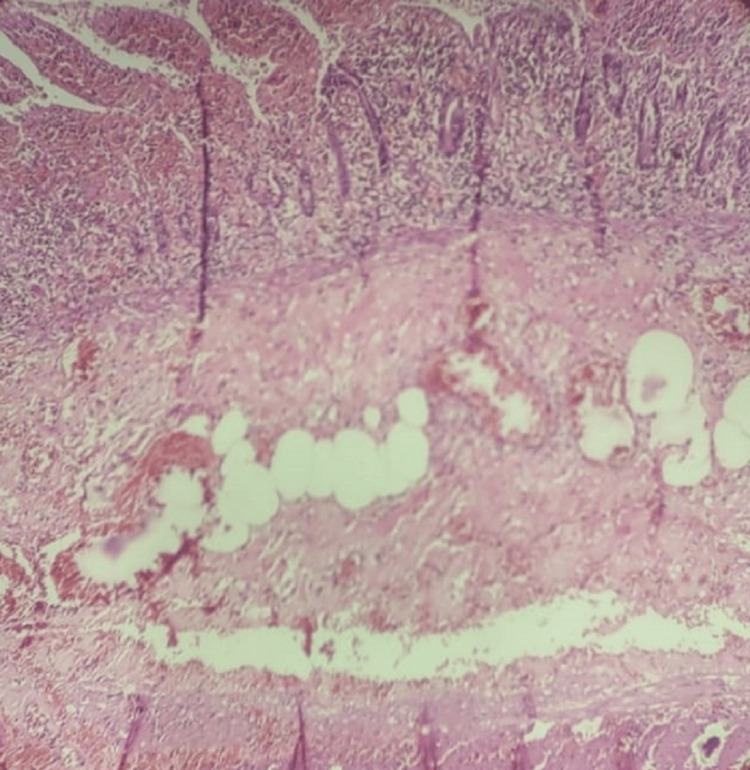
Histopathological features of Meckel's diverticulum with small bowel specimen with sections showing full thickness areas of transmural ischemic necrosis along with numerous congested blood vessels and hemorrhage.

The abdomen was then closed in layers. Histopathological examination of the specimen revealed that the sections from the diverticulum showed full-thickness areas of transmural ischemic necrosis, along with numerous congested blood vessels and hemorrhage, as seen in Figure 5.

The patient was initiated with sips of water on the fourth postoperative day. The patient's recovery after surgery went without any complications, and they received their discharge from the hospital on the ninth postoperative day. The patient was reviewed after two weeks and one month and was uneventful.

## Discussion

Congenital abnormalities of the small intestine are rather frequent, with MD being the most incidental during procedures like autopsy, laparotomy, or barium studies [10]. Although this is prevalent [11], it is often undetected and its occurrence decreases with age. MD is typically identified in a relatively small number of individuals, around 4%, who might have effects such as bleeding, perforation, inflammation, or small bowel obstruction [12]. The most typical presentation of MD includes gastrointestinal bleeding, brought on by acid-induced ulceration in the diverticulum with the gastric mucosa. The other common presentation is intestinal obstruction, which occurs due to volvulus or inflammation surrounding the affected MD. Differentiation between MD and normal small bowel on computed tomography (CT) can be difficult in uncomplicated circumstances. Despite this, the presence of a blind-ending fluid or gas-filled structure related to the small bowel could support our diagnosis [13]. Arteriography and technetium pertechnetate scanning are useful for diagnosing significant bleeding or the presence of ectopic gastric mucosa [8]. In most cases, diagnostic laparoscopy is the preferred and more reliable method, as in our case. It is crucial to exercise caution as delayed treatment can result in significant morbidity and mortality. The occurrence rate of diverticulitis and perforation is approximately 20%, and both conditions are frequently unrecognizable from acute appendicitis up until they are encountered on the table during surgery [14]. Diagnosis of MD prior to surgery can be challenging due to the complex nature of this disorder, which might appear similar to various other intra-abdominal disorders that may be present, including acute appendicitis, inflammatory bowel disease, or other factors that might induce small bowel obstruction [15].

The predominant obstruction observed was intussusception, with the MD serving as the leading cause. Additional complications include volvulus occurring around fibrous bands that are attached to the umbilicus, as well as inflammatory adhesions, Littre's hernias, and diverticular strictures [14]. The axial rotation of the MD is an unusual presentation, which is due to the twisting of the MD on its axial axis. Gangrene of MD, resulting from axial torsion, as presented in this study, is an exceedingly uncommon occurrence, with less than 10 documents available in the literature, thereby proving the rarity of this condition. The onset of gangrene in the MD, resulting from axial torsion around its base, has been associated with its attachment with either the umbilicus or the ileal mesentery. Anatomically, in particular, the length and diameter of the MD are significant contributing factors [16]. The size of the diverticulum might vary, although it usually appears as a short and wide opening (with a mean length of 2.9 cm and width of 1.9 cm) [17]. A long and slender MD with a constricted neck is more prone to torsion, while short, wide MDs are susceptible to foreign body entrapment [18]. The size of the MD in the case we have presented had a length of 8 cm and a breadth of 1.9 cm, with signs of hemorrhagic infarction in histopathology, showing that the long MDs are prone to torsion with respect to anatomy [16]. Obstruction is more prevalent in cases of a large MD [13]. Torsion of a giant MD can be incomplete and recurrent leading to subacute intestinal obstruction. There is also looping of a part of the bowel - ileum in our case - on the base of the MD, thereby paving for the intestinal obstruction in the patient. Less than four documented cases have been noted on this, owing to the rarity of the presentation of this case. The extrusion of the terminal ileum into the loop of MD may have caused it to rotate axially, which in turn caused an impairment in blood supply and led to gangrene of the MD [12].

MD can be treated via open or laparoscopic techniques. The surgical options available include a diverticulectomy, which is a procedure to remove the diverticulum, or ileal resection and anastomosis [6]. As in our case, we proceeded with Meckel's diverticulectomy, which involved resection and anastomosis of the small bowel in an end-to-end, double-layered fashion. The histopathology of the specimen revealed features consistent with MD, along with evidence of hemorrhagic infarction.

## Conclusions

A significant number of MD cases are asymptomatic and appear as a small stump in the distal ileum, often with a minor or absent fibrous band. When symptomatic, it is important to include complications such as obstruction, bleeding, or diverticulitis as potential causes. These should always be included in our list of differentials. These conditions are infrequently identified before surgery due to their similar characteristics to other abdominal disorders. Inflamed diverticula are sometimes discovered inadvertently. They are often found as inflamed diverticulum, found incidentally, and very rarely they are encountered gangrenous, especially due to axial rotation of the MD with ileal knotting at the base leading to sub-acute obstruction owing to the rarity of presentation of this case. Owing to the prompt presentation and proper management, the conventional treatment for MD was established. Else would have been extensive, with significant morbidity and mortality.

## References

[1] Park JJ, Wolff BG, Tollefson MK, Walsh EE, Larson DR (2005). Meckel diverticulum: the Mayo Clinic experience with 1476 patients (1950-2002). Ann Surg.

[2] Opitz JM, Schultka R, Göbbel L (2006). Meckel on developmental pathology. Am J Med Genet A.

[3] Sagar J, Kumar V, Shah DK (2006). Meckel's diverticulum: a systematic review. J R Soc Med.

[4] Wong CS, Dupley L, Varia HN, Golka D, Linn T (2017). Meckel's diverticulitis: a rare entity of Meckel's diverticulum. J Surg Case Rep.

[5] Agha RA, Franchi T, Sohrabi C, Mathew G, Kerwan A (2020). The SCARE 2020 guideline: updating consensus Surgical Case Report (SCARE) guidelines. Int J Surg.

[6] Blouhos K, Boulas KA, Tsalis K (2018). Meckel's diverticulum in adults: surgical concerns. Front Surg.

[7] St-Vil D, Brandt ML, Panic S, Bensoussan AL, Blanchard H (1991). Meckel’s diverticulum in children: a 20-year review. J Pediatr Surg.

[8] Rami Reddy SR, Cappell MS (2017). A systematic review of the clinical presentation, diagnosis, and treatment of small bowel obstruction. Curr Gastroenterol Rep.

[9] Malhotra S, Roth DA, Gouge TH, Hofstetter SR, Sidhu G, Newman E (1998). Gangrene of Meckel's diverticulum secondary to axial torsion: a rare complication. Am J Gastroenterol.

[10] Lüdtke FE, Mende V, Köhler H, Lepsien G (1989). Incidence and frequency or complications and management of Meckel’s diverticulum. Surg Gynecol Obstet.

[11] Cartanese C, Petitti T, Marinelli E, Pignatelli A, Martignetti D, Zuccarino M, Ferrozzi L (2011). Intestinal obstruction caused by torsed gangrenous Meckel's diverticulum encircling terminal ileum. World J Gastrointest Surg.

[12] Sharma RK, Jain VK, Kamboj S, Murari K (2008). Gangrenous Meckel's diverticulum causing acute intestinal obstruction in an adult. ANZ J Surg.

[13] Elsayes KM, Menias CO, Harvin HJ, Francis IR (2007). Imaging manifestations of Meckel's diverticulum. AJR Am J Roentgenol.

[14] Dumper J, Mackenzie S, Mitchell P, Sutherland F, Quan ML, Mew D (2006). Complications of Meckel's diverticula in adults. Can J Surg.

[15] You JS, Chung SP, Park YS, Yu JS, Park YA (2007). A case of strangulated small bowel obstruction caused by Meckel's diverticulum in an adult. J Emerg Med.

[16] Tan YM, Zheng ZX (2005). Recurrent torsion of a giant Meckel's diverticulum. Dig Dis Sci.

[17] Limas C, Seretis K, Soultanidis C, Anagnostoulis S (2006). Axial torsion and gangrene of a giant Meckel’s diverticulum. J Gastrointestin Liver Dis.

[18] Bani-Hani KE, Shatnawi NJ (2004). Meckel's diverticulum: comparison of incidental and symptomatic cases. World J Surg.

